# A Multispectroscopic Study of 3d Orbitals in Cobalt Carboxylates: The High Sensitivity of 2p3d Resonant X-ray Emission Spectroscopy to the Ligand Field**

**DOI:** 10.1002/anie.201204855

**Published:** 2012-12-06

**Authors:** Matti M van Schooneveld, Robert W Gosselink, Tamara M Eggenhuisen, Mustafa Al Samarai, Claude Monney, Kejin J Zhou, Thorsten Schmitt, Frank M F de Groot

**Affiliations:** a Inorganic Chemistry and Catalysis, Debye Institute for Nanomaterials Science, Utrecht UniversityUniversiteitsweg 99, 3584 CG Utrecht (The Netherlands); b Swiss Light Source, Paul Scherrer Institut (PSI)5232 Villigen PSI (Switzerland)

**Keywords:** cobalt, ligand field theory, photochemistry, UV/Vis spectroscopy, X-ray emission spectroscopy

Determination of the ligand coordination number and symmetry of a transition metal ion is important to understand reaction mechanisms in inorganic chemistry. The problem can be addressed through diffraction techniques or by spectroscopy. Here we limit ourselves to the latter and note that, historically, the problem has been studied with UV/Vis, or optical absorption or electronic spectroscopy.^1^ Recently however the field of resonant X-ray emission spectroscopy (RXES) developed at a high pace.^2^ Here we show that metal 2p3d RXES is highly sensitive to the metal ion ligand field. We present a comparison of UV/Vis, 2p X-ray absorption spectroscopy (XAS), and 2p3d RXES on a series of cobalt(II) carboxylates. The X-ray data were acquired at the state-of-the-art ADRESS beamline.^3^ We show that 2p XAS and UV/Vis have a limited discriminative power compared to 2p3d RXES. Through ligand field multiplet (LFM) calculations we show that 2p3d RXES allows the most judicious analysis of the ligand field. While previous 2p3d RXES studies on metal oxides revealed its d–d sensitivity,^4^ this is the first such observation on inorganic complexes. More importantly, the notion that 2p3d RXES measures element-selective, more as well as more intense d–d excitations than UV/Vis, and that this allows a more reliable determination of the ligand field, is novel. 2p3d RXES will allow unraveling reaction mechanisms of important 3d-metal-mediated chemical processes.

RXES, also known as resonant inelastic X-ray scattering (RIXS) or resonant Raman X-ray scattering (RRXS), is a synchrotron-based technique commonly used in solid-state physics.2a In RXES the studied material is irradiated with X-rays to excite a core electron (a nonvalence electron) to empty electronic states. The term ‘resonant’ implies excitation at a material-specific core electron binding energy, in contrast to normal or ‘nonresonant’ X-ray emission spectroscopy, where photon energies higher than the binding energy are used. The created core hole is filled by an electron from a higher electron shell. This can occur under emission of a photon (radiatively) and the energies of such photons are measured as a RXES spectrum. Note that the overall RXES process is charge neutral in contrast to, for example, X-ray photoelectron spectroscopy (XPS). Figure 1 depicts the process (in a single-particle view). By X-ray absorption a metal 2p core electron is transferred to an empty metal 3d orbital. When recording the X-ray absorption over a range of empty 3d states one performs 2p or L_2,3_ XAS. With 2p3d RXES the subsequent radiative decay of 3d electrons to the 2p core hole is followed. The final state may then possess an excited 3d state. As such 2p3d RXES presents a close analogy to UV/Vis, in which UV and visible light can be used to excite 3d electrons to empty 3d states (see Figure 1). Effectively, both UV/Vis and 2p3d RXES measure d–d transitions, which are used in ligand field theory to determine the symmetry and coordination number of metal ions. While 2p XAS does not measure d–d transitions, it is also often employed to characterize the ligand field. This shared ligand field sensitivity triggered the herein presented comparison between the three spectroscopies.

**Figure 1:**
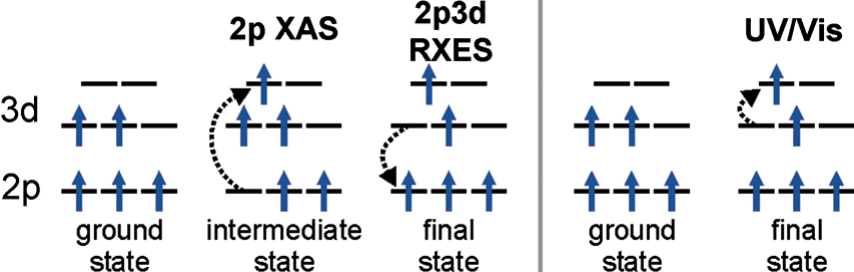
Schematic representations of the photon-induced electron transitions in 2p XAS, 2p3d RXES, and UV/Vis spectroscopy. The 2p3d RXES and UV/Vis final states are identical.

Cobalt(II) diformate (**1**), diacetate (**2**), dibenzoate (**3**), and dioleate (**4**) were obtained commercially and used as model compounds. Such salts in general can attract moist, which may lead to adsorbed water coordinating to the metal ion and to partial carboxylate hydrolysis. Therefore the salts were characterized by X-ray powder diffraction (XRD), atomic absorption spectroscopy (AAS), carbon–hydrogen–nitrogen (CHN) analysis, and thermogravimetric analysis (TGA) as explained in the Supporting Information and Figure S1 therein.^5^ Table 1 summarizes the results. Compound **1** is cobalt(II) diformate dihydrate; **2** is a cobalt(II) diacetate salt that is less hydrated than the fully hydrated tetrahydrate salt; **3** is cobalt(II) dibenzoate; and **4** is likely cobalt(II) dioleate dissolved in excess oleic acid. Compound **1** contains two distinct Co^II^ sites that both are sixfold coordinated in octahedral *O*_h_ symmetry.^6^ One of the sites is tetragonal distorted. Compound **2** may contain Co^II^ with different coordination numbers owing to its partly hydrated nature, but the analysis suggests that on average five oxygen ligand atoms bind. For **3** the maximum coordination number is four and for **4** it is likely four. Despite these uncertainties, the analysis clearly reveals that the average Co^II^ coordination number decreases from **1** till **3**. Such differences should be observable in ligand-field-sensitive spectroscopies. Note that it is not our aim to give a perfect spectroscopic description of pure model compounds, but that we want to illustrate the experimental 2p3d RXES sensitivity to d–d excitations and show that RXES theory correlates these well to the ligand field.

**Table 1:** Characterization of the cobalt(II) carboxylates.

Sample	Molecular formula	Average coordination number Co^II^
**1**	[Co(HCOO)_2_(H_2_O)_2_]	6
**2**	[Co(H_3_CCOO)_2_(H_2_O)_3_]	5
**3**	[Co(H_5_C_6_COO)_2_]	≤4
**4**	[Co(H_33_C_17_COO)_2_]+H_33_C_17_COOH	(4)

Figure 2 shows the 2p_3/2_ XAS, 2p3d RXES, and UV/Vis spectra of **1**–**4**. Figure 2 a shows the XAS spectra in which **2**–**4** cannot be distinguished from each other. The RXES spectra in Figure 2 b exhibit much stronger differences, the clearest appearing at approximately 0.3 eV. No peak is visible for **1**, it is of intermediate intensity in **2**, and it is strong for **3** and **4**. Figure 2 c shows the UV/Vis spectra, which are also discriminative. We will continue by comparing the techniques on a number of experimental issues: ease of acquisition and data statistics, energy resolution, element selectivity, selection rules, and probed energy range. UV/Vis spectroscopy is a standard laboratory technique, whereas XAS and RXES are synchrotron-based, and the number of machines where one can obtain 2p3d RXES spectra is still limited. Moreover, the time needed to collect an RXES spectrum with similar statistics as an XAS spectrum is a factor 10–100 higher. UV/Vis spectroscopy further has a resolution of approximately 1–10 meV full-width-at-half-maximum (fwhm). In 2p_3/2_ XAS the lifetime of the 2p_3/2_ core hole limits the resolution to approximately 400 meV fwhm for the 3d metals. In 2p3d RXES the natural resolution is in principle set by the same 3d-excited final state as in UV/Vis, but the RXES setup limits, until date, the resolution to approximately 100 meV fwhm at the cobalt 2p edge.4e So, if UV/Vis data acquisition is easier and it also has a superior resolution, why should one consider 2p XAS and 2p3d RXES? The first reason is that both XAS and RXES are element-selective, because they are probed at an element-selective core level. In UV/Vis, the presence of different chromophores or metals in a sample often inhibits its spectral interpretation. Owing to the energy selectivity RXES allows also the acquisition of spectra at different excitation energies over the X-ray absorption edge. In Figure 2 a the numbers *a*–*e* indicate five energies at which spectra were acquired in this study. In Figure 2 b the spectra at energy *d* are shown (the others are shown in Figures S2 and S3 in the Supporting Information). The ability to acquire different spectra allows for a higher level of characterization and is an advantage of RXES over both UV/Vis and XAS. Considering the selection rules, 2p XAS and 2p3d RXES present additional advantages. They involve one or two electric-dipole-allowed electron transitions, respectively. Therefore, in 2p3d RXES, d–d transitions are not parity- or dipole-forbidden, while they are in UV/Vis. This implies that 2p3d RXES has a higher sensitivity to d–d transitions. Also, the conservation of the dipole-selection rule facilitates the interpretation of the X-ray spectra by theory. When it comes to the spin-selection rule, in UV/Vis the electron spin must in principle be unchanged during a transition. In case of 2p XAS and 2p3d RXES, the strong 2p core hole spin-orbit coupling permits the spin-selection rule to be alleviated.4a,b This implies that spin-forbidden d–d transitions are more visible in 2p3d RXES than in UV/Vis.

**Figure 2:**
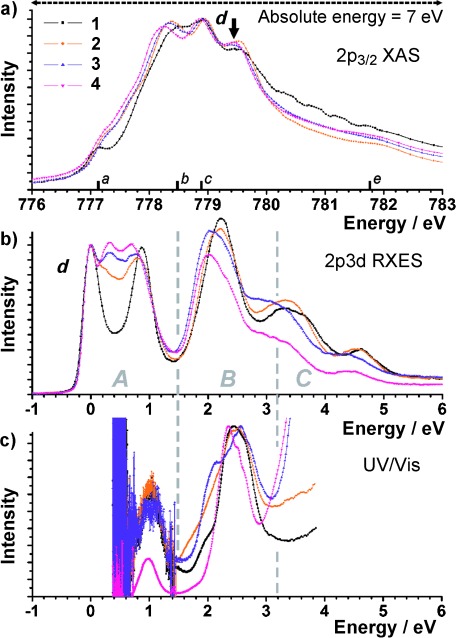
a) Cobalt 2p_3/2_ XAS, b) 2p3d RXES at excitation energy *d*, and c) UV/Vis spectra of 1–4. The legend in (a) applies to the whole figure. Regions *A*, *B*, and *C* are indicated. The numbers *a*–*e* indicate five energies at which spectra were acquired in this study.

Figure 2 reveals that 2p3d RXES and UV/Vis both probe excitations in the energy window that comprises the empty valence 3d states, while 2p XAS shows the empty valence 3d states modified by the 2p core hole presence. Figure 2 c further shows that UV/Vis spectroscopy in the region below 1.5–1 eV is inhibited. As the terminology implies, UV/Vis is not well-suited for the (near) infrared domain where vibrational modes of the material shadow the detection of electronic transitions. We call the region where this occurs region *A* to facilitate a more detailed comparison between UV/Vis and 2p3d RXES later on. We note that the exact border of the energetic region is somewhat arbitrary set to 1.5 eV. This was done for an additional, technical reason that prevents observance of low-energy features: most standard laboratory UV/Vis spectrometers do not measure below 1.5 eV (for Figure 2 c we used an advanced spectrometer). In Figure 2 b it is revealed that metal d–d excitations in region *A* are however adequately probed by 2p3d RXES. This is a result of the element selectivity of RXES. In addition, we introduced regions *B* and *C* in Figure 2 b, c based on the UV/Vis spectra of compounds **1**–**4**. The division has a spectroscopic nature, since we use it to divide that part of the UV/Vis spectrum that is dominated by metal d–d excitations (region *B*) from the part dominated by ligand-to-metal or metal-to-ligand charge-transfer (CT) transitions (region *C*). Since the energy at which CT starts to occur is material-dependent, the region border is equally so. In practice, CT transitions for most inorganic complexes occur above 3 eV. The reason that CT transitions shadow d–d excitations is that only the latter are dipole-forbidden. This results in typical molar extinction coefficients ε of approximately 10^2^ and 10^4^ L mol^−1^ cm^−1^ for the d–d and CT transitions, respectively. In 2p3d RXES, in contrast, the d–d transitions are not forbidden and there is no reason that they should be less intense. In fact, for transition metal oxides with divalent metal ions (for example MnO, CoO, and CuO) CT transitions are experimentally observed to be much less intense than d–d transitions and their 2p3d RXES spectra can be well interpreted without CT effects.4c–e We ascribe this to the localized nature of the 2p to 3d excitation, plus, for the divalent metal ions, the small extent of 3d-electron delocalization on to the oxygen atoms. For Co^II^ in **1** to **4** we observe in the RXES spectra taken at excitation energy *d* also only little influence of CT. This can be seen by comparing with spectra taken at exciation energy *e* (see Figure 2a and Figures S2, S3 in the Supporting Information). The satellite in the XAS spectrum at which these spectra were measured, is mainly due to CT transitions and this strongly enhances the CT effects in the RXES spectra.^7^ The comparison shows that the peaks around approximately 4 eV gain relative intensity and that an extra peak occurs at approximately 5 eV. However, even at this CT-sensitive energy, the spectrum is dominated by d–d excitations. In general however it remains to be quantified by future studies what the 2p3d RXES sensitivity is to CT, especially for higher metal valencies. On a technical base we note that most UV/Vis spectrometers do not measure above 5 eV, while 2p3d RXES data can easily be measured up to 20 eV. It can now be understood that 2p3d RXES probes d–d transitions in regions *A*–*C* with a high sensitivity, while UV/Vis reveals such transitions only in region *B* owing to their shadowing by vibrational modes in region *A* and CT transitions in region *C*.

As a short intermezzo we point out that the spectral features of **1**–**4** occur at higher energies in UV/Vis than in 2p3d RXES. The shifts are quantified in Figure S4 in the Supporting Information. The error in the absolute energy of the RXES spectra is ≤100 meV and ≤10 meV in the UV/Vis spectra. This cannot explain the observed shifts of ≥150 meV. In the Supporting Information we present ideas to investigate this further, but it is beyond the current scope to determine the origin(s) of the shifts.

Until now we made an experimental comparison that revealed the high 2p3d RXES sensitivity to d–d transitions. In the Supporting Information we summarize how ligand or crystal field theory^1^ is used to determine the ligand coordination number of a transition metal ion and its symmetry from spectra. Below we explain why region *A* in 2p3d RXES spectra is especially sensitive to the ligand field. Figure 3 shows the experimental 2p_3/2_ XAS spectrum and 2p3d RXES spectrum at excitation energy *d* of compound **1**. Furthermore an LFM theoretical interpretation is shown; calculated with CTM4XAS.^8^ The energies of atomic manifolds of the XAS and RXES spectra are shown as sticks (or “barcodes”). When a ligand field is applied, the atomic manifolds are split in multiple ligand field manifolds each over typically a range of a few eV.^9^ Figure 3 a reveals that the density of ligand field manifolds in a 2p XAS spectrum is easily approximately 10–100 eV. Given that the experimental resolution is only approximately 0.4 eV fwhm, peaks cannot be ascribed to single manifolds. This makes it difficult to relate an experimental spectral shape to one set of ligand field parameters on a purely spectroscopic basis. In 2p3d RXES the situation is more orderly, since in general the ligand field manifold density is approximately 2–20 eV and the spectral resolution is approximately 0.1 eV fwhm. Especially in region *A* the manifold density is low, which implies that experimental peaks consist of few manifolds.^10^ In addition, the manifolds may stem from one atomic manifold as indicated in Figure 3 b for weak ligand fields.^11^ The above does not mean that a 2p3d RXES spectrum can now be described by one set of ligand field parameters. The number of observed peaks and their energies merely restrict the choice of parameters to symmetries that can reproduce these numbers and energies. These are however much clearer restrictions than provided by 2p XAS, where peak energies and numbers depend on more than the ligand field.

**Figure 3:**
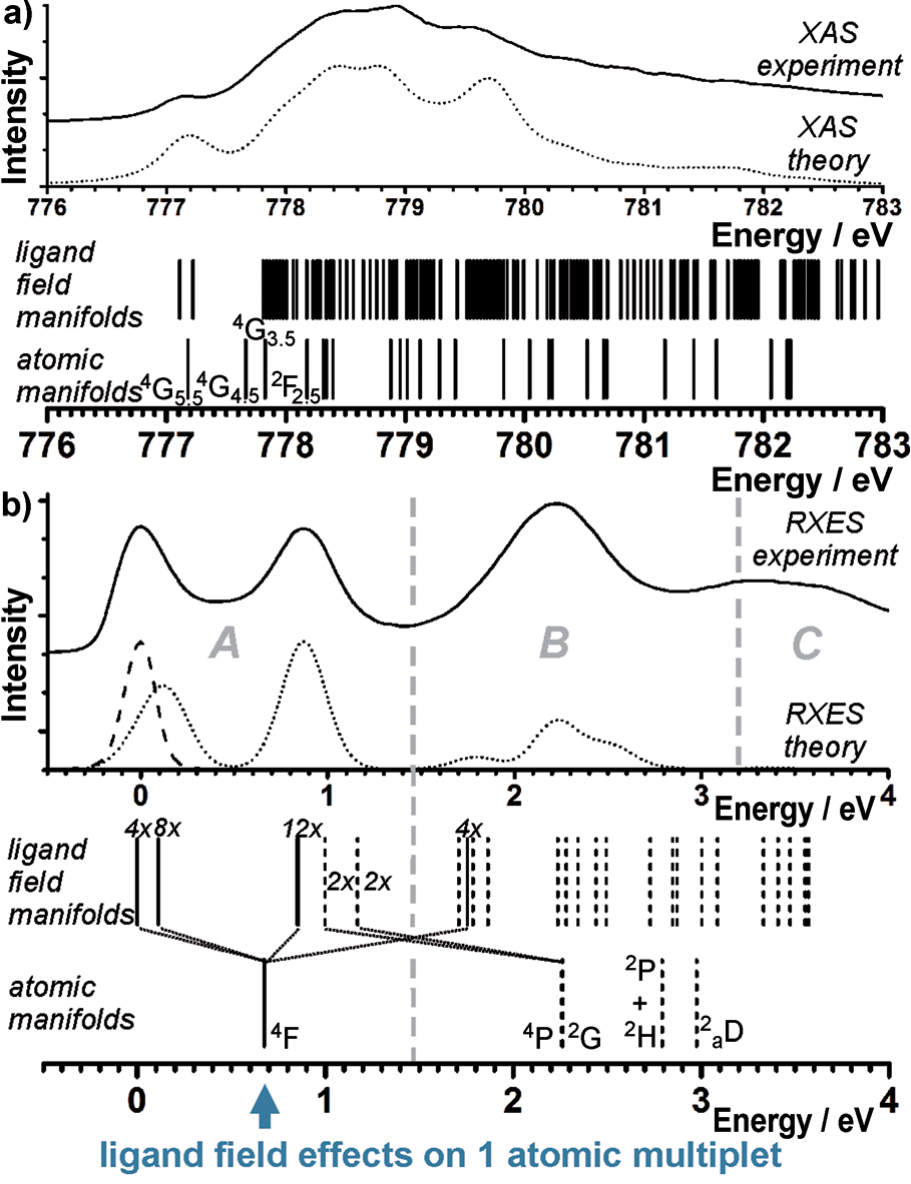
Experimental (solid) and LFM (dotted) a) 2p_3/2_ XAS and b) 2p3d RXES at excitation energy *d* of 1. The dashed spectrum at 0 eV was taken on a non-cobalt reference sample to reveal background X-ray scattering. The manifold energies are given as sticks. In (b) the atomic ^4^F and its ligand field split manifolds are shown as solid sticks. The numbers (e.g. 4×) indicate state degeneracies.

Determination of the ligand field from UV/Vis spectra is more difficult. To our knowledge, no ab initio methods exist that simulate full UV/Vis spectra of transition metal compounds quantitatively. Even calculation of only d–d excitations (neglecting CT and other light-absorbing parts of a molecule), causes problems because of breaking of the dipole-selection rule. Most calculations that do exist use time-dependent density functional theory (TD-DFT), but these yield only one-electron excitations.^12^ In practice, determination of the ligand field from UV/Vis data is done with Tanabe–Sugano diagrams.^13^ Such diagrams are best known for 3d^*n*^ configurations in *O*_h_ symmetry, but also exist for lower symmetries. Consider now the UV/Vis spectra in Figure 2 c. Each compound has two main peaks: at approximately 1 and 2.5 eV. If the ligand field symmetry is determined from this, it should be identified in Tanabe–Sugano diagrams of all symmetries, if the ratio of these peak energies occurs in these diagrams. This would result in many possible symmetries. Moreover, one would likely conclude that **1**–**4** possess an equivalent symmetry. This would be wrong given that the RXES spectra show a peak at approximately 0.3 eV for **3** and **4**. Moreover, the fine structure in region *B* of the UV/Vis data would not facilitate the assignment. At these energies many ligand field manifolds exist that all mix and form the peak at approximately 2.5 eV (see Figure 3 b). What would restrict the number of solutions from UV/Vis data, is that the molar absorption coefficient ε depends in an established way on symmetry. For example Co^II^ in tetrahedral *T_d_* symmetry is more dipole-allowed than in *O*_h_ symmetry, because the former is not centrosymmetric. Such relationships remain to be established for 2p3d RXES. In theory however, assignment based on intensities should be easier with 2p3d RXES, because LFM calculations are quantitative.

Figure 4 shows the experimental 2p3d RXES spectra at excitation energy *d* of **1**–**4** compared with their LFM spectra. In all calculations the Co^II^ is high-spin and the used LFM parameters are given in the Supporting Information. The spectra are calculated in a tetragonal *D*_4*h*_ symmetry where we find reasonable theoretical spectra if we keep the crystal field parameter Dt at 0 eV for all compounds. The most important differences when simulating the spectra of **1**–**4** are the decrease of 10Dq from 0.9 to 0.6 eV and the increase of Ds from 0.05 to 0.15 eV. These values correspond to a slightly distorted octahedral *O*_h_ symmetry for **1** and a tetragonal *D*_4*h*_ symmetry for **4**. Single-particle representations of the 3d-level energies and the way they depend on 10Dq and Ds are given in Figure 4. Sketches of the ligand coordination are also given for both *O_h_* and *D*_4*h*_ symmetry. For **1** and **4** the ligand field manifolds that stem from the atomic ^4^F ground state manifold are given as solid sticks above or below the respective spectra. The ground state ligand field manifold is ^4^A_2g_(^4^F) for all compounds. The ^4^E_g_←^4^A_2g_ transition^14^ is increased from 0.11 to 0.32 eV when going from **1** to **4**. This transition gives rise to the peak that is present at approximately 0.3 eV in compounds **3** and **4**. Simultaneously, the peak consisting of ^4^E_g_(^4^F)+^4^B_2g_(^4^F)←^4^A_2g_(^4^F) transitions at 0.87 eV decreases to 0.73 eV when going from **1** to **4**. This behavior is a direct result of the lowering of the symmetry from distorted *O*_h_ to *D*_4*h*_. The peak at approximately 2.5 eV consists of many transitions, but includes the ^4^E_g_(^4^P)+^4^A_2g_(^4^P)←^4^A_2g_(^4^F) transitions for all compounds. Since these are spin-allowed, this also explains why the peak at approximately 2.5 eV is relatively intense in the UV/Vis spectra. Finally, the symmetry is related to the coordination number of the metal ion, and the gradual change from distorted *O*_h_ to *D*_4*h*_ in **1**–**4** is in good agreement with the lowering of the coordination number as given in Table 1. LFM XAS spectra calculated with the same parameters, and details on the LFM RXES, are given in Figures S5–S8 in the Supporting Information. We also put our results in the context of four alternative d–d sensitive techniques in the Supporting Information.^15^

**Figure 4:**
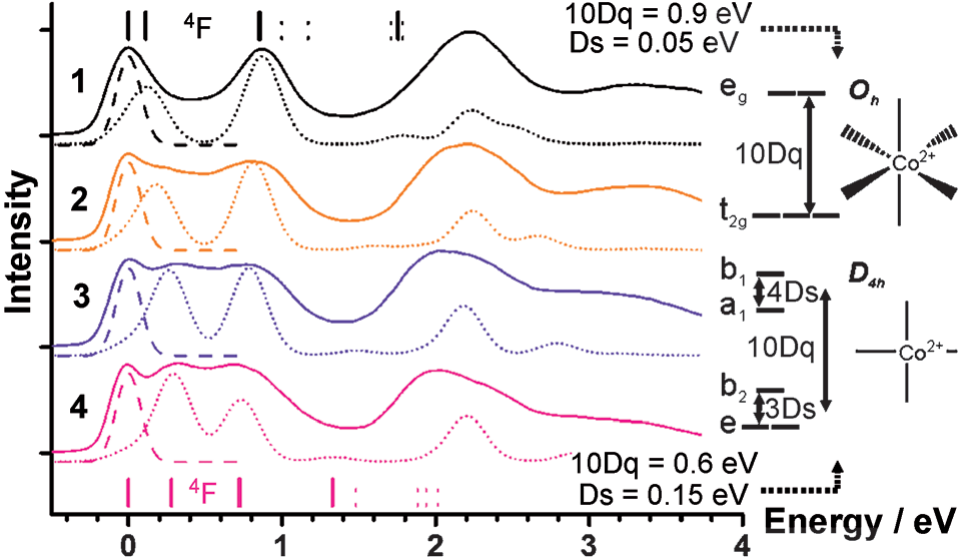
The same RXES spectra (solid) as shown in Figure 2 b together with their LFM spectra (dotted). The dashed spectrum at 0 eV was taken on a non-cobalt reference sample. Energies of the ligand field manifolds below 2 eV are given as sticks for 1 and 4. Solid sticks originate from the ^4^F atomic multiplet; dotted sticks from other atomic multiplets. To the right schemes of the 3d-level energies and the Co^II^ coordination in *O*_h_ and *D*_4*h*_ symmetry are shown. Dq and Ds are crystal field parameters.

In summary, a multispectroscopic investigation of metal 2p XAS, metal 2p3d RXES, and UV/Vis was carried out on a set of cobalt(II) carboxylates. We show that 2p3d RXES is able to discern the different complexes, while 2p XAS does not. In addition, 2p3d RXES detects element-selective, more as well as more intense d–d excitations in transition metal systems than UV/Vis. On the basis of ligand field multiplet calculations we explain why 2p3d RXES allows the most judicious determination of ligand field parameters, compared to 2p XAS or UV/Vis. Finally, we deduce ligand field parameters from the 2p3d RXES spectra of the cobalt(II) carboxylates and deduce coordination numbers from them. We find good agreement with coordination numbers found by independent techniques. Overall, we foresee that d–d sensitive 2p3d RXES holds great promise for the study of 3d-metal-mediated chemistry. It is our hope that renewable energy research, including solar fuel chemistry and catalysis, will find its way to the technique. Given the increasing scarcity of heavier metals, the urgency for 3d-metal sensitive techniques becomes even more evident.

## Notes

Detailed facts of importance to specialist readers are published as ”Supporting Information”. Such documents are peer-reviewed, but not copy-edited or typeset. They are made available as submitted by the authors.
