# Supplementary material for: A Multispectroscopic Study of 3d Orbitals in Cobalt Carboxylates: The High Sensitivity of 2p3d Resonant X-ray Emission Spectroscopy to the Ligand Field
Source: Angew Chem Int Ed Engl. 2012 Dec 6;52(4):1170–4. doi: 10.1002/anie.201204855 (PMC3564409; doi:10.1002/anie.201204855)
Supplement: Supplementary file 1 [file anie0052-1170-sd1.pdf]

Angewandte Chemie
Eine Zeitschrift der Gesellschaft Deutscher Chemiker

Supporting Information

© Wiley-VCH 2013

69451 Weinheim, Germany

A Multispectroscopic Study of 3d Orbitals in Cobalt Carboxylates: The High Sensitivity of 2p3d Resonant X-ray Emission Spectroscopy to the Ligand Field**

Matti M. van Schooneveld, Robert W. Gosselink, Tamara M. Eggenhuisen, Mustafa Al Samarai, Claude Monney, Kejin J. Zhou, Thorsten Schmitt, and Frank M. F. de Groot**

anie_201204855_sm_miscellaneous_information.pdf

Supporting Information

Table of contents

Cobalt(II) carboxylate characterization S2
- Experimental section S2
- Results and discussion S3

2p XAS, *2p3d* RXES and UV/Vis measurements S6
- Experimental section S6
- Results S7
  - *2p* XAS spectra S7
  - *2p3d* RXES spectra S7
  - *2p3d* RXES versus UV/Vis spectra S8

2p XAS and *2p3d* RXES ligand field multiplet (LFM) calculations S9
- Ligand field theory for spectra S9
- Computational section S10
- Results and discussion S13
  - *2p* XAS spectra S13
  - The *2p3d* RXES plane S14
  - Temperature effects on *2p3d* RXES spectra S15
  - Symmetry labels of the 3*d*-states S16
  - Comparison of *2p3d* RXES and UV/Vis S18

Alternative d-d sensitive techniques S19

References S20

Cobalt(II) carboxylate characterization

Experimental section

Materials and tools. Cobalt(II) diformate, dibenzoate and dioleate (≥94%) were purchased from City Chemical LLC. Cobalt(II) diacetate (99.995% trace metals basis, ≤5% water) was purchased from Sigma-Aldrich. The cobalt(II) carboxylates were stored in a nitrogen-atmosphere glove box, analyzed under inert atmospheres and used as received unless otherwise stated. An airtight XRD specimen holder with dome-like X-ray transparent cap to protect air-sensitive materials (A100B33; sample reception 25 mm diameter, 1 mm depth) was obtained from Bruker AXS. As explained in the main text, the cobalt(II) diformate, diacetate, dibenzoate and dioleate are referred to as compound ***(1)-(4)***, respectively.

Atomic absorption spectroscopy (AAS), CHN-analysis, thermogravimetric analysis (TGA) and X-ray powder diffraction (XRD). The cobalt content and the carbon and hydrogen content were determined by AAS and CHN-analysis respectively on the as-obtained compounds ***(1)-(4)*** by the Mikroanalytisches Laboratorium Kolbe. The analysis was done under exclusion of air in an argon atmosphere to prevent compound hydration (further than already present in the as-obtained compounds). TGA was performed on a Q50 TA Instrument to determine the temperature-dependent mass losses upon heating compounds ***(1)-(4)*** under a stream of nitrogen gas. To this end, the sample masses were measured while heating the samples to 500 °C with a heating rate of 5 °C/min and subsequently during another 30 min while keeping the sample at 500 °C. TGA samples were prepared inside a nitrogen-atmosphere glove box and the used cups for analysis were closed inside the glove box to prevent hydration (again: further than already present in the as-obtained compounds). XRD diffraction patterns were acquired on a Bruker-AXS D8 advance diffractometer. Cobalt $K\alpha_{1,2}$ (λ=1.790 Å) radiation was used from a X-ray tube operated at 30 kV and a current of 45 mA. Typically, data points were acquired between 5°<2θ<62° every 0.017° with 1 s $step^{-1}$. XRD samples of the as received cobalt(II) carboxylates were prepared inside a nitrogen-atmosphere glove box and enclosed in an airtight and X-ray transparent box to probe the as received powders.

Results and discussion

(1)-(4) were characterized because such compounds may in general adsorb water from the atmosphere. The water may bind at the metal ion site and/or lead to partial carboxylate hydrolysis. This would than change the symmetry and coordination number of the metal ion. Since we aim to study the correlation between *2p3d* RXES spectroscopy and the metal ion ligand field it is important to verify the metal coordination sphere with independent techniques.

XRD was performed on samples ***(1)-(3)*** since these are solid at room temperature (***(4)*** is a liquid at room temperature). Database matching of the obtained diffractograms revealed ***(1)*** to be cobalt(II) diformate dihydrate ([$Co(HCOO)_2.2H_2O$]) as shown in the top panel of Figure S1. For compounds ***(2)*** and ***(3)*** no database matches were found, but a reference XRD pattern was measured for cobalt(II) diacetate tetrahydrate, which is the fully hydrated form of cobalt(II) diacetate, and this was significantly different from the diffractogram of ***(2)***.

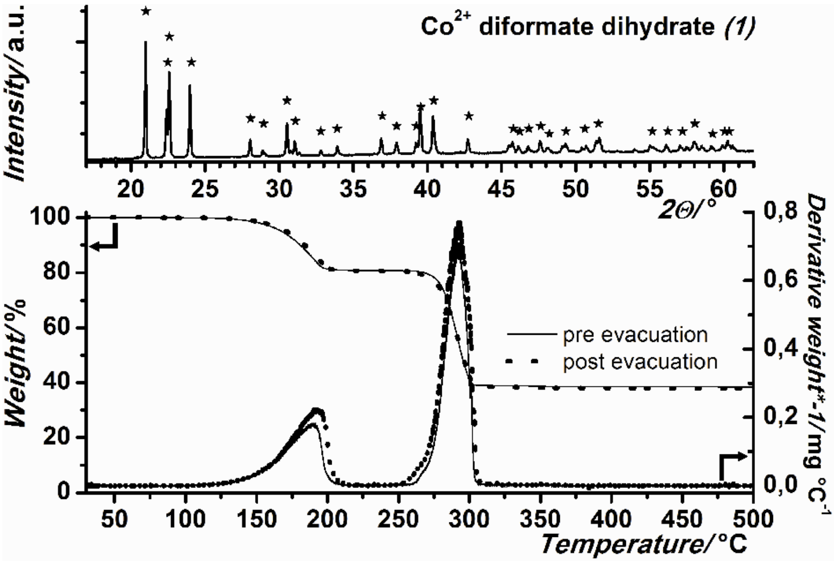

Figure S1. Top: The powder X-ray diffractogram of compound ***(1)*** matches the black stars indicating the peak maxima of the reference diffractogram of cobalt(II) diformate dihydrate. Bottom: TGA curves of ***(1)*** and their derivatives before (solid black line) and after (dotted black line) evacuation to ~10^{-6} mbar for two hours.

Subsequently, all compounds were investigated with an AAS spectrometer and CHN analyzer to determine the cobalt content and the carbon plus hydrogen content, respectively. The data revealed the molar ratios as indicated in the second and third column of Table S1. From these ratios the number of carboxylate groups per cobalt ion was obtained, by assuming that all carbon was present in the carboxylate groups. All excess hydrogen (moles of hydrogen not covered for by the carboxylate groups) was assumed to be present in water molecules. This is a rough assumption, since the hydrogen could also be present in for example hydroxyl ligands, but the current analysis does not allow for a further discrimination. The fourth column in Table S1 gives the tentative molecular formulae based on these assumptions.

Table S1. Element and TGA analysis of compounds ***(1)*** to ***(4)***.

	AAS and CHN-analysis			*TGA*				
**Sample**	**Mole C/ mole Co**	**Mole H/ mole Co**	**Molecular formula[a]**	**Mass loss (%)**	**Rest mass (%)[b]**	**Mole H_2O/ mole Co**	**Molecular formula[c]**	**Average coordination number Co^{II}[d]**
(1)	2.0	5.8	$[Co(HCOO)_{2.0}1.9H_2O]$	61	39	2.2	$[Co(HCOO)_{2.0}2.2H_2O]$	6
(2)	3.8	12.0	$[Co(H_3C_2OO)_{1.9}3.1H_2O]$	68	32	3.2	$[Co(H_3C_2OO)_{1.9}3.2H_2O]$	5
(3)	12.3	11.5	$[Co(H_5C_7OO)_{1.8}1.4H_2O]$	73	27	0.0	$[Co(H_5C_7OO)_{1.8}]$	≤4
(4)	51.9	-	$[Co(H_{33}C_{18}OO)_{2.9}?H_2O]$	91	9	0.0	$[Co(H_{33}C_{18}OO)_{2.0}]$+0.9*$H_{33}C_{18}OOH$	(4)

[a] Assumptions: all carbon is present in the carboxylate groups and the excess of hydrogen is present in water. [b] Assumption: rest mass is only due to Co_3O_4. [c] Assumption: the carbon/cobalt ratio from the AAS/CHN analysis is used. [d] See the text for a discussion on the average Co^{II} coordination numbers.

TGA analysis of ***(1)-(4)*** was then used to test these assignments. The fifth and sixth column of Table S1 show the overall mass loss and the rest mass percentages of ***(1)-(4)*** as determined by TGA. In addition, for ***(1)*** the TGA curve is shown in the bottom panel of Figure S1. Two separate mass losses of 19% at ~175 °C and of 42% at ~285 °C could be identified. Overall 61% of weight is lost as shown in the fifth column of Table S1. For the analysis we assume that all cobalt in the complexes was converted to Co_3O_4, or in other words, that the rest mass is Co_3O_4 only. This assumption is common practice, even when working in a nitrogen gas flow: upon starting a TGA measurement the closed sample cup gets perforated which does expose it from that moment on to air. From the determined amount of Co_3O_4 the molar cobalt content in the original compound is calculated. The mass loss is due to the non-cobalt content. We use the cobalt to carboxylate ratios as determined by AAS/CHN analysis to calculate what part of the mass loss stems from the loss of carboxylates. The remaining part of the mass loss is ascribed to loss of water as given in the seventh column of Table S1. This is thus an estimation of the water content in addition to the one based on AAS/CHN analysis. Since the hydrogen determination in the CHN analysis had for example a 0.5% standard deviation over 3.2% of hydrogen mass in compound ***(1)*** we consider the TGA data to be more reliant and use these in our final assignment of the compound molecular formulae.

Based on the above, the mass losses for compound ***(1)*** at ~175 °C and at ~285 °C as shown in the TGA curves in Figure S1, are ascribed to 2.2 moles of water and 2.0 moles of formate, respectively. For compound ***(2)*** to ***(4)*** we do not give the temperature-dependent decomposition profiles, but only show the tentative molecular formulae in column eight of Table S1. For compound ***(2)*** the formula could be described as cobalt(II) diacetate trihydrate. We emphasize that (in light of the stated assumptions) this is merely an estimation of the molecular formula, but we point out that it is ruled out on the basis of this analysis that the salt is in its fully hydrated tetrahydrate form, as was also clear from XRD analysis. Compound ***(3)*** may on the basis of the element analysis be thought to be partly hydrated, but the mass loss in the TGA corresponds to the loss of two benzoate groups only and we assign the compound to be cobalt(II) dibenzoate. For ***(4)*** both element and TGA analysis match with an average molecular formula of cobalt(II) trioleate. Since, to the best of our knowledge, cobalt(II) trioleate does not exist, we assume the sample to consist of cobalt(II) dioleate dissolved in additional oleic acid.

Some conclusions can now be drawn on the average cobalt coordination numbers in ***(1)-(4)***. These are shown in the ninth column of Table S1. From crystallographic studies it is known that ***(1)*** has a monoclinic structure with a $P2_{1/c}$ space group. It possesses two distinct Co^{II} sites, both with six oxygen atoms around the Co^{II} in an octahedral arrangement. In one site the six oxygen atoms all come from formate ions, while in the second site four oxygen atoms are from water molecules and two from formate ions. The two differently coordinated cobalt ions are bridged by one of the formate ions. Due to the two different ligands around the Co^{II} ions in the latter site, this site has a distorted octahedral coordination.[5]

For ***(2)*** the crystal structure is unknown, but it is known for the fully hydrated cobalt(II) diacetate tetrahydrate. This is also a monoclinic structure with a $P2_{1/c}$ space group. Here the Co^{II} ions are all in an octahedral environment surrounded by four oxygen atoms from four water molecules and two oxygen

atoms from two different acetates.[15a] Since compound ***(2)*** was estimated to contain three instead of four water molecules per cobalt we estimate the average coordination number to be 5. It may however well be that this sample contains multiple Co^{II} sites that are for example 4-, 5- and 6-fold coordinated. For ***(3)*** a maximum of four oxygen carboxylate atoms can bound per Co^{II} ion, which gives a maximum coordination number of 4. In absence of a crystal structure the actual coordination number remains however unknown and could also be lower. For compound ***(4)*** our analysis is inconclusive, but we consider a coordination number of four most likely in case of a cobalt(II) dioleate. Overall, we note that the more hydrophilic the carboxylate group is, the more water was estimated to be present in the structures, leading to a larger average coordination number.

In addition to the compound structural characterization it was verified that such compounds could stand high vacuums, since the X-ray measurements are performed at ~10^{-8} mbar. To this end, compound ***(1)*** (being the most hydrated compound) was evacuated in a separate experiment to ~10^{-6} mbar for two hours and measured again with TGA thereafter. This measurement, also shown in the bottom panel of Figure S1, revealed that the hydration state of the cobalt(II) diformate dihydrate was unaltered due to the low pressure exposure.

2p XAS, *2p3d* RXES and UV/Vis measurements

Experimental section

XAS and RXES Tools. p-Type Boron-doped silicon (100)(111) surface terminated wafers (525 µm thick; cut in 7x7 mm squares; resistivity ~ 5 Ωcm) were obtained from CrysTec GmbH. Hydrofluoric acid (pro analysis, 48-51%) was obtained from Acros Organics. Plastic tweezers (style KR) were obtained from Rubis Switzerland. Sticky carbon tape was obtained from NEM Nisshin Em.Co.Ltd. Silver epoxy paste (E4110 kit) was obtained from Epoxy Technology.

Sample preparation for XAS and RXES. The solids ***(1)-(3)*** were pressed into sticky carbon tape for XAS and RXES measurements. The liquid ***(4)*** was dripped onto silicon wafers. Prior to this the silicon substrates were treated to remove oxygen species from the surface and to passivate the wafers with atomic hydrogen. The wafers were immersed respectively in acetone, 2-propanol and deionized water and given an ultrasonic treatment for 10 min each. The substrates were etched for 10 min with a 1/5/4 v/v/v solution of HF/ethanol/water.[4e] Subsequently, the wafers were rinsed with deionized water to remove physisorbed surface species before drop casting ***(4)*** on them.[4e] The wafers were attached with sticky carbon tape to an aluminum holder designed to fit the synchrotron set-up. Silver epoxy paste between the substrate and transfer tool improved conductivity. All was done inside a glove box with helium-atmosphere. The holder was introduced in an inert environment to the load lock chamber of the ADRESS beamline, which was then pumped down to ~10^{-8} mbar before sample transfer into the XAS/RXES analysis chamber. The authors thank the Swiss spallation neutron source SINQ, Paul Scherrer Institute, Switzerland to allow them to work in their chemistry laboratory.

XAS and RXES spectra acquisition and treatment. Total fluorescence yield (TFY) XAS spectra were acquired using a photodiode close by the samples. The spectra were sampled with 25 points eV^{-1}. The RXES spectra were measured using the high-resolution Super Advanced X-ray Emission Spectrometer (SAXES) at the ADRESS beamline of the Swiss Light Source (SLS).[3] A scattering geometry was used in which the angle between the incoming light vector and the outgoing one was 90°. The incoming light was polarized linear parallel (depolarized geometry or linear horizontal; LH) to the scattering plane with a grazing incident angle of 20°. Measuring at grazing incidence was done to minimize the self-absorption of the elastic (Rayleigh) peak. Measuring at LH polarization was done to increase the cross-section of the resonant features as compared to the elastic peak. By positioning the beam at a non-cobalt-containing and amorphous reference and varying the incoming energy from 770-780 eV in steps of 1 eV $step^{-1}$, the elastic peak was used to calibrate the RXES detector. For this purpose the elastic peaks were fitted with Gaussian functions and the maxima of the fits were plotted against detector-channels. The plot was fitted with a linear curve to determine the energy-channel response function. At the cobalt $2p_{3/2}$ XAS edge (~780 eV) the combined energy resolution of the RXES zero-loss peak was 196 meV fwhm. In order to obtain resonant spectra the incident energy was tuned to and over the cobalt *2p* XAS edge. Spectra were sampled with 52 points eV^{-1}, and summed over 4-10 partial spectra, which were acquired for 10 min each. In order to compare the RXES spectra, they were normalized to the intensity of the elastic peak (in Figure 2b) or to the peak with the maximum intensity (Figure S3). The pressure in the analysis chamber was ~10^{-8} mbar during all measurements and the spectra were acquired at 22±5 °C. For both XAS and RXES measurements we did not notice any evolution of the spectra during the experiment.

Ultraviolet-visible (UV/Vis) spectroscopy. Diffuse reflectance UV/Vis spectra of compound ***(1)-(3)*** and transmission UV/Vis spectra of compound ***(4)*** were acquired on a Perkin-Elmer 950 spectrometer between λ =3300-330 nm with 2 nm $step^{-1}$.

Results

Here we give the full cobalt *2p* XAS spectra, indicate all excitation energies for the *2p3d* RXES spectra (including an additional energy *f* that was not shown in Figure 2a), give all *2p3d* RXES spectra and give the experimental comparison between the *2p3d* RXES and UV/Vis spectra.

2p XAS spectra

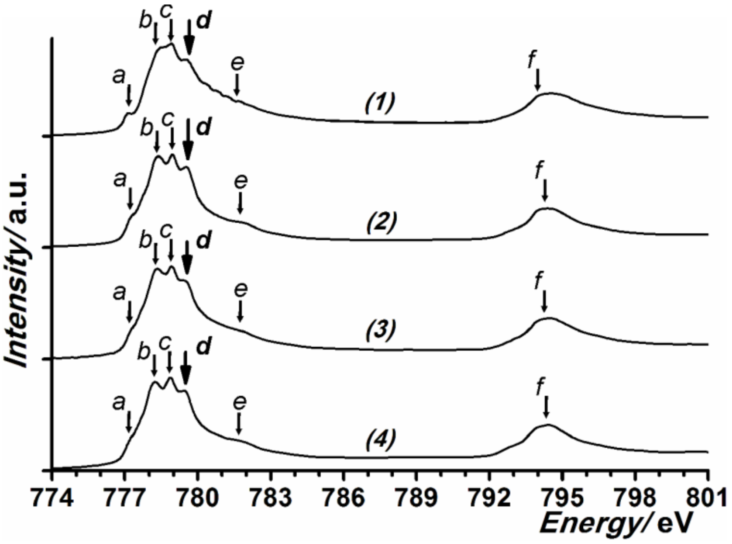

Figure S2. TFY cobalt *2p XAS* spectra of compound ***(1)*** to ***(4)***. The excitation energies *a-f* at which *2p3d* RXES spectra were acquired are indicated.

2p3d RXES spectra

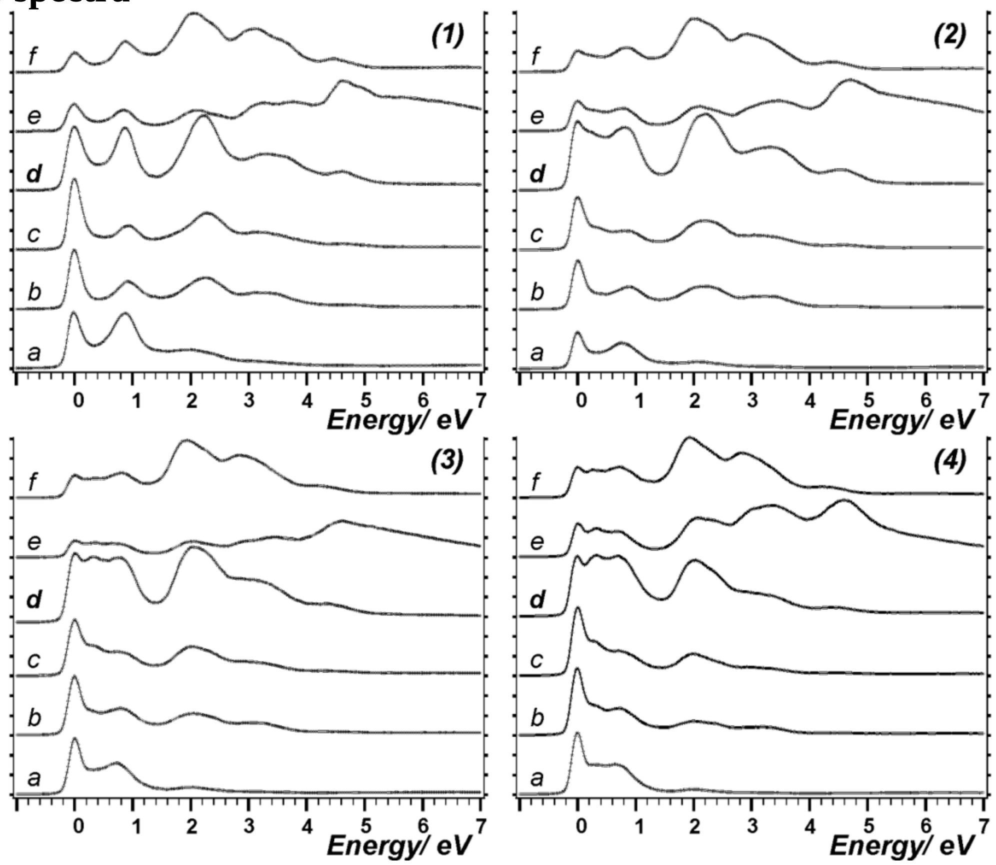

Figure S3. Experimental cobalt *2p3d* RXES spectra of compound ***(1)*** to ***(4)***. The spectra were measured in the linear horizontal (LH) polarized geometry at excitation energies *a-f* as indicated in Figure S2.

2p3d RXES versus UV/Vis spectra

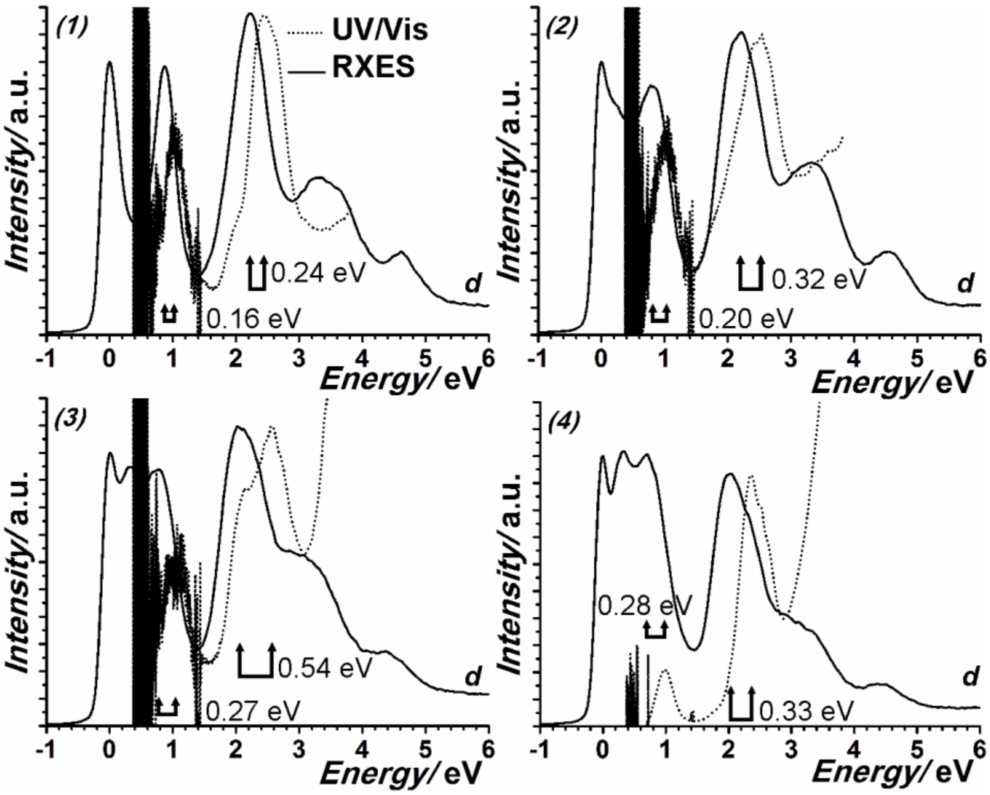

Figure S4. Cobalt *2p3d* RXES spectra at excitation energy *d* (solid lines) and UV/Vis spectra (dotted lines) of compound ***(1)*** to ***(4)***. The arrows indicate energy differences between peaks measured by RXES and UV/Vis and the actual differences are given. The thick black bands in the UV/Vis spectra around 0.5 eV are due to vibrational modes of air compounds.

2p XAS and *2p3d* RXES ligand field multiplet (LFM) calculations

Ligand field theory for spectra

How to determine the ligand coordination number of a transition metal ion and its symmetry from spectra? This question is traditionally addressed by ligand or crystal field theory and we refer to excellent accounts that deal with the topic.[1] Here we briefly repeat how this theory is used to interpret spectra.

In classical LFM theory, electronic spectra of transition metal compounds are simulated on the basis of a single transition metal ion in a potential field created by the ligands. It is realized that spectral features of the transition metal-related structure of such compounds, in the first place, depend on intra-atomic electron effects of the metal ion itself. Their result is that, for a constant electron population of d-orbitals (for example a $3d^7$ configuration), many ways exist to arrange the electrons over the orbitals. Each state has its own energies. Groups of degenerate atomic states are called atomic multiplets or manifolds. Transitions between different manifolds are called d-d transitions. In second place, the atomic manifolds are affected by the ligand field. Its main effect is to split the atomic manifolds in states with different energies. These states can again form degenerate subgroups that may be labeled with symmetry labels specific to the ligand field. In the semi-empirical determination of the ligand field symmetry of a transition metal ion from a spectrum, one tries to determine what kind of symmetry may perturbate the atomic manifolds in such a way that they give rise to the observed experimental spectra. Through the obtained symmetry of the ligand field one subsequently determines the metal ion coordination number.

We use ligand field multiplet (LFM) theory that is employed in the freeware program CTM4XAS to interpret our spectra.[7] A detailed explanation of its functioning and the input parameters used in the calculations is given in the next section. In short, *2p* XAS spectra depend on more atomic effects than *2p3d* RXES spectra. This is a result of the 2p core hole in the final XAS state and its interactions with 3d electrons. As a consequence, the number of atomic multiplets is larger in the XAS final state than in the RXES final state (the only exception is the $2p^5 3d^{10}$ XAS versus $3d^9$ RXES final state). For a $2p^5 3d^n$ configuration in general 6x10!/(10-n)!n! atomic states exist where *n* is the number of d-electrons. For a $3d^n$ configuration this number is 10!/(10-n)!n!. For Co^{II} this implies that the XAS and RXES final state consist of respectively 270 and 120 atomic states. This gives rise to 45 respectively 10 manifolds with different energies. The same numbers of possible XAS and RXES dipole-allowed transitions with different energies exist and effectively the final states are the constituents of the spectra. In ligand field theory the atomic states are subsequently further split by the ligand field.

In theory, the description of a spectrum by more different manifolds (*2p* XAS versus *2p3d* RXES) implies that the spectrum can be better defined. However, given (I) the density of ligand field manifolds in a *2p* XAS spectrum and (II) the experimental resolution, this does not hold in practice as discussed in the main text.

Computational section

Ligand field multiplet (LFM) calculations. *2p* XAS and *2p3d* RXES spectra were simulated using the freeware multiplet program available under the name Charge Transfer Multiplet for XAS (CTM4XAS).[7a] The splitting of d-levels due to atomic effects are calculated using a Hartree-Fock (HF) code that includes relativistic effects as developed by Cowan.[7b, 7c] This generates the atomic multiplets or atomic manifolds. The further splitting of atomic multiplets in a ligand field and/or due to charge transfer effects can subsequently be calculated in CTM4XAS using the Butler-Thole codes.[7d] For the current calculations only the ligand field effects were included, since CT effects modify the *2p* XAS and *2p3d* RXES spectra of compounds ***(1)*** to ***(4)*** only little (as discussed in the main text). In such cases we consider the omission of CT effects in the calculations to be favorable. The less semi-empirical parameters needed to simulate the spectra, the more physical meaning the results get. The values of the applied electronic structure parameters used in the interpretation are summarized in Table S2.

Table S2. Energetic values in eV of the electronic structure parameters used in the LFM calculations for respectively compound ***(1)***/***(2)***/***(3)***/***(4)***. Where single values are given these apply to all compounds.

Co^{II} con figuration	Initial state $2p^63d^7$	Intermediate state $2p^53d^8$	Final state $2p^63d^8\underline{d}$
F^2_{dd}	7.891/8.356/8.356/8.820	8.430/8.925/8.925/9.421	7.891/8.356/8.356/8.820
F^4_{dd}	4.902/5.190/5.190/5.479	5.242/5.550/5.550/5.858	4.902/5.190/5.190/5.479
F^2_{pd}	-	4.937/5.227/5.227/5.518	-
G^1_{pd}	-	3.670/3.886/3.886/4.102	-
G^3_{pd}	-	2.087/2.210/2.210/2.333	-
ζ_{3d}	0.050	0.062	0.050
ζ_{2p}	-	9.748	-
10Dq	0.900/0.800/0.700/0.600	0.900/0.800/0.700/0.600	0.900/0.800/0.700/0.600
Ds	0.050/0.080/0.120/0.150	0.050/0.080/0.120/0.150	0.050/0.080/0.120/0.150
Γ (fwhm)	-	0.400 (*2p*$_{3/2}$)/0.800 (*2p*$_{1/2}$)	0.020 (*3d*)
G (fwhm)	-	0.200	0.200
B[a]	0.105/0.112/0.112/0.118	0.113/0.119/0.119/0.126	0.105/0.112/0.112/0.118
C	0.389/0.412/0.412/0.435	0.416/0.440/0.440/0.465	0.389/0.412/0.412/0.435
β	0.871/0.929/0.929/0.978	-	0.871/0.929/0.929/0.978

[a] Strictly speaking Racah parameters B and C and the nephelauxetic parameter β are no LFM input parameters, but they follow directly out of F^2_{dd}/F^4_{dd} through relations given in the text and are shown to facilitate the comparison with UV/Vis.

LFM calculations for XAS. For our calculations we approximate cobalt to have a formal Co^{II} valence in compound ***(1)*** to ***(4)***. Co^{II} can be described effectively by a $2p^63d^7$ ground state configuration. For simulation of the *2p* XAS spectra all photon-excited electric dipole-allowed electron transitions from the ground state to a $2p^53d^8$ excited state are calculated, together with their oscillator strengths, in intermediate coupling. To this end, the ground state and excited state energy levels are first calculated.

The atomic multiplets in the ground state depend mainly on the 3d-3d Coulomb interactions and 3d spin-orbit coupling ζ_{3d}. The excited state is in addition affected by 2p-3d Coulomb and exchange interactions and 2p spin-orbit coupling ζ_{2p}. The 3d-3d Coulomb and 2p-3d Coulomb and exchange interactions can be described by two-electron integrals, called the Slater or Slater-Condon integrals. The radial parts of these integrals are obtained *ab initio* within the Hartree-Fock limit for a given electron configuration by the program. The program also automatically scales the values to 80% of their original values to correct for intra-atomic configuration interaction and hybridization effects. The radial parts of the integrals that define the 3d-3d Coulomb interactions are F^2_{dd} and F^4_{dd} and are given in Table S2. In an analogous way the 2p-3d Coulomb interactions are set by F^2_{pd} and the 2p-3d exchange interactions by G^1_{pd} and G^3_{pd}. Next to the Couloumb and exchange interactions the program calculates the 2p and 3p spin-orbit coupling values within the Hartree-Fock limit, but it does not scale the original values further down.

The user can modify the obtained atomic parameters in order to correct for effects not included in the Hamiltonian. Here we have further reduced both the values of the radial parts of the Slater integrals and the value of the 3d spin-orbit coupling. The Slater parameters (F^2_{dd}, F^4_{dd}, F^2_{pd}, G^1_{pd} and G^3_{pd}) were all

scaled simultaneously to one percentage per compound. When going from ***(1)*** to ***(4)*** these percentages were 85, 90, 90 and 95% respectively (this corresponds to 68, 72, 72 and 76% of the HF values). Reduction of the Slater integrals, in particular the ones describing the 3d electrostatic interactions (F^2_{dd} and F^4_{dd}), is often done in LFM calculations to effectively describe the delocalization of 3d electrons on to ligands. As such it implicitly corrects for effects of charge transfer. This d-electron delocalization is also known as the nephelauxetic effect.[1b] In fact, F^2_{dd} and F^4_{dd} are related to the Racah parameters B and C through relations given by Cowan[7c]: $B=(9F^2_{dd} - 5F^4_{dd})/441$ and $C=5F^4_{dd}/63$. The nephelauxetic parameter β is than defined as $B_{compound}/B_{free\ ion}$. We used the free ion value of B = 971 cm^{-1} for Co^{II}.[12] Overall, this implies that, when going from ***(1)*** to ***(4)*** the d-electrons are slightly more localized on the metal ion. Furthermore, the value of ζ_{3d} was reduced here to 75% of its atomic Hartree-Fock value. We will not discuss the reason for this reduction for we have previously done so for Co^{II} in CoO on page 11 of Van Schooneveld et al.[4e]

Once the atomic multiplets are calculated, a further splitting or branching of these states occurs in the ligand field. Here the D_{4h} point group symmetry is used to simulate the effect of the carboxylate ligands. In D_{4h} the ligand field is described by the parameters 10Dq, Ds and Dt. The values of 10Dq and Ds are given in Table S2, while Dt was 0 eV in all our calculations. Without Dt we could sufficiently reproduce the experimental spectra.

The transitions that are calculated between the ground and excited, ligand-field-effected, atomic states yield absolute intensities. These intensities of the discrete transitions are convoluted with Lorentzian functions $\Gamma_{2p_{3/2}}$ or $\Gamma_{2p_{1/2}}$ to account for the $2p_{3/2}$ or $2p_{1/2}$ core hole lifetime broadening at the $2p_{3/2}$ or $2p_{1/2}$ XAS edge, respectively. A second convolution of the allowed transitions is done with a Gaussian function G (fwhm) to account for the instrumental broadening. Note that the calculations do not yield absolute energy positions and the resulting spectra are shifted in energy for comparison with the experimental spectra. All XAS spectra were calculated at a temperature of 22 °C.

The different states of the initial state configuration $2p^63d^7$ have been labeled in Table S3 in D_{4h} symmetry without 3d spin-orbit coupling using the following method: in spherical symmetry without 3d spin-orbit coupling the energy level degeneracies, Tanabe-Sugano linear formulas and Hund's rules were used to assign symmetry labels. Progressively an octahedral ligand field O_h was introduced by increasing 10Dq in steps of 0.3 eV, a tetragonal distortion D_{4h} was introduced in steps of 0.05 eV and finally a 3d spin-orbit coupling was applied in steps of 25% of its atomic Hartree-Fock values. During the introduction of these parameters the labeled states were systematically tracked and the results were (as far as possible) in agreement with the work of Griffith.[1a]

LFM calculations for 2p3d RXES. The inelastic scattering function F(Ω,ω) is described by the resonant term of the Kramers-Heisenberg formula[2d] that defines the interference between the X-ray induced electron excitation and subsequent decay processes:

$$F(\Omega,\omega)=\sum_j \left| \sum_i \frac{<f|T_2|i><i|T_1|g>}{E_g+\hbar\Omega-E_i+\mathrm{i}\Gamma_i} \right|^2 \times \frac{\Gamma_f/2\pi}{(E_g+\hbar\Omega-E_f-\hbar\omega)^2+\Gamma_f{}^2/4}$$

where |g>, |i> and |f> indicate the ground, intermediate and final state of the probed matter and E_g, E_i, E_f are the energies. Here ħΩ and ħω are the energies of the incident and emitted photon. T_1 and T_2 are operators describing the radiative transitions by absorbed and emitted photons and are electric dipole in nature for the *2p3d* RXES transitions. Γ_i gives the spectral Lorentzian broadening due to the finite time of the core-hole existence in the intermediate state. The second term implies that energy should be conserved in the overall RXES process and that a further Lorentzian broadening Γ_f occurs due the finite life time of the hole in the final state.

The LFM simulations of the *2p3d* RXES spectra for Co^{II} are done using the $g=2p^63d^7 \rightarrow i=2p^53d^8 \rightarrow f=2p^63d^8\underline{d}$ transitions. Although the final state $2p^63d^8\underline{d}$ is formally equal to $2p^63d^7$ we use this notation to indicate that the final state still can have a hole in the 3d band. Only in an elastic scattering event, where

$\hbar\Omega = \hbar\omega$, the final state has no 3d hole (other than the initial three 3d holes). Lorentzian functions accounting for the intermediate ($\Gamma 2p_{3/2}$ or $\Gamma 2p_{1/2}$) and final ($\Gamma 3d$) state lifetime of the hole were used to convolute the allowed RXES transitions. Gaussian functions accounting for monochromator- and spectrometer-induced experimental broadening were applied to convolute the spectra further. All RXES calculations took the interference effects between the X-ray absorption and emission into account. The shown RXES calculations were all done for the ground state, which formally relates to a situation at -273.15 °C (zero Kelvin). Only in Figure S7 calculations are shown that take the Boltzmann-distributed population of excited states at 22 °C into account. All spectra were calculated with the transition probabilities that correspond to the used LH polarization in the experiment (as discussed in the spectral acquisition part). Therefore the parallel or σ contribution of the light was taken incident on the sample together with the perpendicular or π contribution being detected (in the CTM4XAS program this corresponds to “parallel” incident light plus outgoing “left” light summed with “parallel” incident plus outgoing “right” light).

Results and discussion

2p XAS spectra

The LFM calculations were able to reproduce all *2p* XAS spectra as shown in Figure S5.

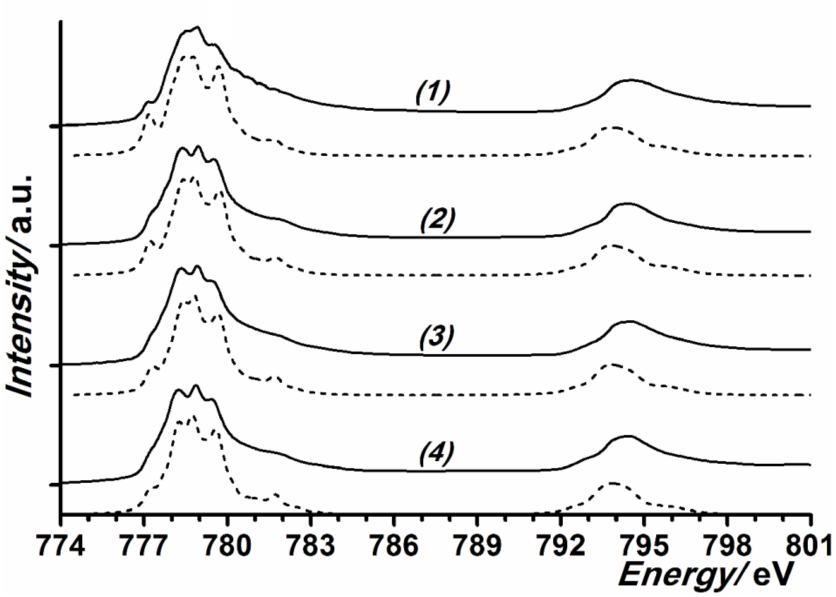

Figure S5. TFY cobalt *2p* XAS spectra (solid lines) of compound ***(1)*** to ***(4)*** as shown in Figure S2 together with their LFM simulations (dotted lines).

The *2p3d* RXES plane

If one measures many RXES spectra over the X-ray absorption edge at a fine interval, one can construct a so-called RXES plane. This is done by making a two-dimensional (2D) contour plot of the RXES intensities as a function of the excitation energy (see for the details for example Glatzel et al.[2d]). One can also calculate such a plane using the CTM4XAS codes. This allows the comparison of theoretical and experimental RXES spectra at every excitation energy over the XAS edge. Figure S6 shows the LFM *2p3d* RXES planes at the cobalt $2p_{3/2}$ and $2p_{1/2}$ XAS edges, for compound ***(1)*** and ***(4)***, and a comparison between the LFM and experimental spectra at excitation energies *a*, *c* and *f* . An increasing number of theoretical and experimental spectral matches increases the reliability of the values found for the LFM input parameters.

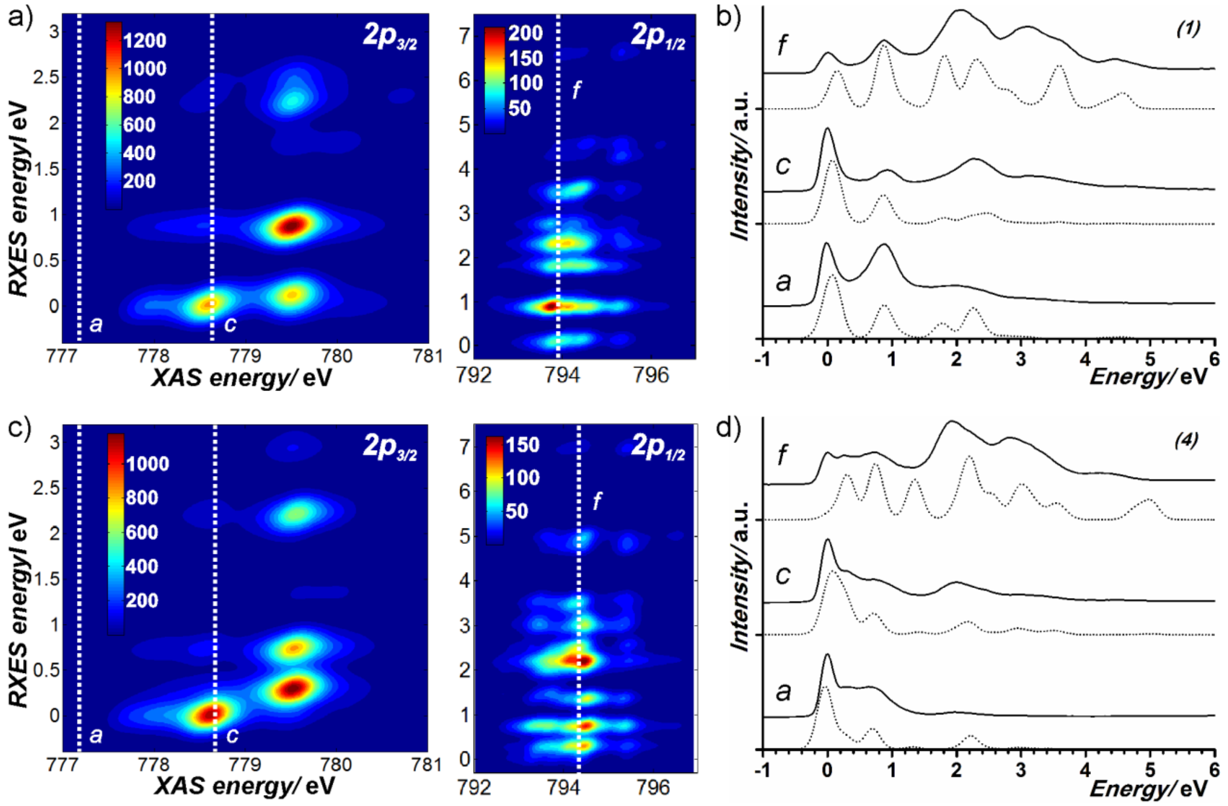

Figure S6. a) LFM *2p3d* RXES planes at both the cobalt $2p_{3/2}$ and $2p_{1/2}$ XAS edge using the parameters found to describe ***(1)***. b) Experimental (solid lines) and theoretical (dotted lines) RXES spectra at excitation energies *a*, *c*, and *f* for ***(1)***. The positions of the spectral cross-sections at these energies are shown as white dotted lines in a). c) The same calculations as shown in a) but now using the parameters describing compound ***(4)***. d) Idem as b) but for ***(4)***.

Temperature effects on *2p3d* RXES spectra

The RXES spectral shape depends on the population of excited states as a function of temperature through the Boltzmann distribution. The occupied fraction of a state p_i is given by $p_i = \exp[(-\Delta E_i/k_b T)/\Sigma_j \exp(-\Delta E_j/k_b T)]$ where k_b is the Boltzmann constant, ΔE_j is the energy of the excited state relative to the ground state and T is the temperature. However, our LFM approach does only take a limited set of physical interactions into account, as explained in the main text and computational section. Phonons, or lattice vibrations, possess low energies (typically below 0.1 eV) and may create a series of (multi)phonon states below 0.5 eV. These may couple with other excitations to increase their energy.[15b] Coupling of vibrations to the electronic states will also influence the overall symmetry of a state, which influences the transition probabilities between states. Charge transfer dependent temperature effects may also occur.[15c] These effects may certainly influence the spectral shape on the given energy scales, but are not calculated here. Despite this, one can calculate the effect of excited state population for the states that are calculated in the LFM approach. Figure S7 shows such calculations for T=22 °C. It follows that only the first excited state is significantly populated and that inclusion of this state has a minor effect on the RXES spectral shape. Therefore the calculations shown in the article constitute of the ground states only. For compound ***(1)-(4)*** the first excited states lay respectively 0.014/0.011/0.010/0.009 eV above ground state. The energies of the second excited states lay respectively 0.103/0.199/0.265/0.293 eV above ground state.

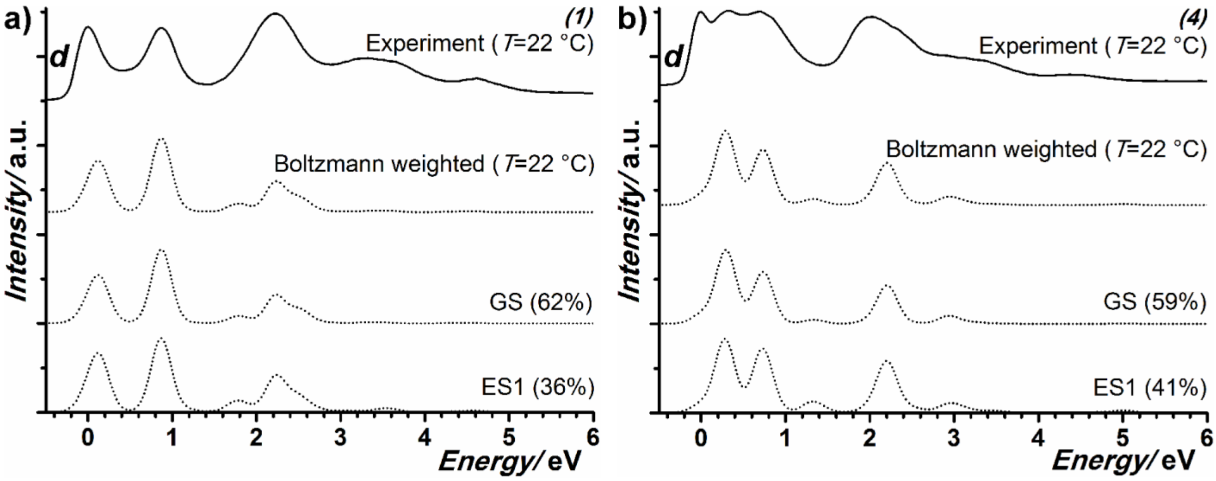

Figure S7. a) Experimental *2p3d* RXES spectrum (solid line) at excitation energy *d* in compound ***(1)***, together with the LFM spectra (dotted lines) of the ground state (GS) and first excited state (ES1). Their spectral weight at 22 °C is indicated in parenthesis. The Boltzmann weighted spectrum is shown. b) Analogous experimental and LFM spectra as shown in a) for compound ***(4)***.

Symmetry labels of the *3d*-states

Figure S8 shows the same experimental *2p3d* RXES spectra at excitation energy *d* of ***(1)*** to ***(4)*** as were given in Figure 4. Six energetic trends are indicated with labels **I-VI**. These trends are based on the LFM calculated spectra of the compounds. More precisely: they follow six peaks that are visible in the convoluted LFM RXES spectra.

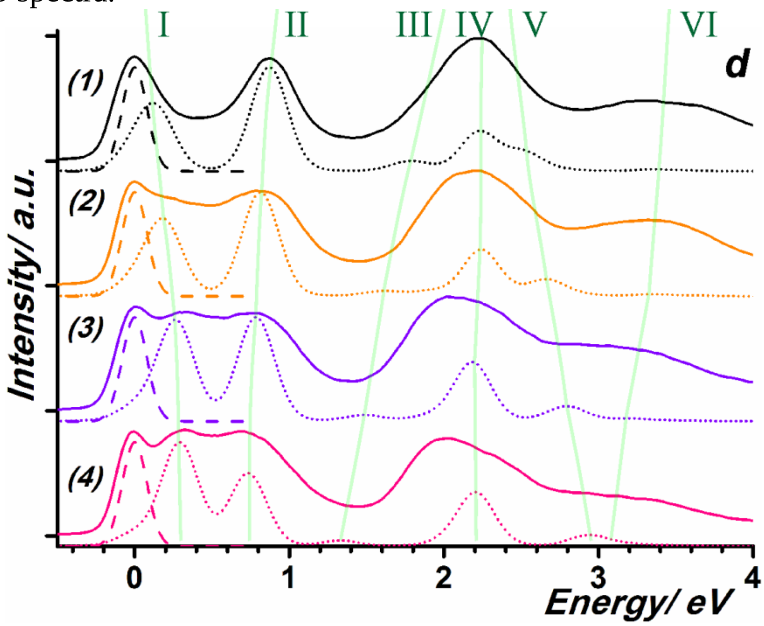

Figure S8. The same RXES spectra (solid lines) as shown in Figure 4 for ***(1)-(4)*** together with their LFM spectra (dotted lines). The dashed lines peaking at 0 eV are experimental spectra taken off the sample.

A comparison between the experimental UV/Vis and RXES energies, together with the LFM RXES interpretation is given in Table S3. For each sample three columns are indicated. In the first and second column the energies of experimental UV/Vis and RXES peaks are given. The third column gives the energies of the convoluted LFM RXES peaks, hence the energies of features **I-VI** in Figure S8. The convoluted LFM peaks consist of many different discrete transitions from the ground to excited state ligand field manifolds. It is beyond the scope of this paper to give all the discrete energies of all calculations. We will give one example of this for compound ***(4)***. Peak **I** in Table S3 is caused by convoluted $^4E_g(^4F)$ states with a peak maximum at 0.29 eV. The discrete states themselves occur at 0.28, 0.29, 0.29 and 0.30 eV. For peak **II**, given in Table S3 at 0.73 eV, the discrete states are at 0.70, 0.72, 0.74, 0.74, 0.75 and 0.77 eV. These stem from $^4E_g(^4F)$ and $^4B_{2g}(^4F)$ manifolds. At higher energies much more ligand field manifolds exist and peak labeling becomes more complex. The energies that we just mentioned are based on the full calculations with the parameters given in Table S1. This implies that ζ_{3d} (3d spin-orbit coupling) is included.

The final column of Table S3 gives the assignment of the LFM convoluted peaks without the ζ_{3d}. The reason for this is that, if we were to include ζ_{3d} the number of symmetry labels would increase even more, which would not be very helpful for our current discussion. Instead we give the symmetry labels in a pure tetragonal D_{4h} symmetry. The atomic manifold from which a ligand field manifold stems, is indicated in parenthesis. As mentioned in the main text, the ground state ligand field manifold is $^4A_{2g}(^4F)$ for all compounds. Peak **I** thus consists for all compounds of $^4E_g(^4F) \leftarrow {}^4A_{2g}(^4F)$ transitions. When going from ***(1)*** to ***(4)*** the convoluted energy of these transitions is increased from 0.11 to 0.32 eV. Peak **II** consist mainly of $^4E_g(^4F)+^4B_{2g}(^4F) \leftarrow {}^4A_{2g}(^4F)$ transitions and its energy goes from 0.87 to 0.73 eV. Peak **III** consists mainly of $^4B_{1g}(^4F) \leftarrow {}^4A_{2g}(^4F)$ transitions and its energy is lowered from 1.79 to 1.33 eV. Peak **IV** consists of many transitions, but includes the $^4E_g(^4P)+^4A_{2g}(^4P) \leftarrow {}^4A_{2g}(^4F)$ transitions. In Table S3 it can be seen that additional ligand field manifolds occur at peak **II** and **III** for compound ***(1)*** and ***(2)***. These are doublet states from the ^2G atomic manifold and move to higher energies for compound ***(3)*** and ***(4)***.

Table S3. Energies in eV of the experimental UV/Vis and RXES spectroscopic features, together with the energies of the LFM convoluted and discrete RXES states of compound ***(1)*** to ***(4)***.

Feature	*(1)* experiment UV/Vis	*(1)* experiment RXES	*(1)* theory LFM convoluted states	*(2)* experiment UV/Vis	*(2)* experiment RXES	*(2)* theory LFM convoluted states	Assignment in D_{4h} without ζ_{3d}
I	-	-	0.12	-	0.25	0.18	$^4E_g(^4F)$
II	1.03	0.87	0.87	1.04	0.80	0.81	$^4E_g(^4F)+^4B_{2g}(^4F)$ $[+^2A_{1g}(^2G)+^2B_{1g}(^2G)]$
III	1.97	-	1.79	-	-	1.62	$^4B_{1g}(^4F)$ $[+^2E_g(^2G)+^2A_{2g}(^2G)+^2E_g(^2G)+^2B_{2g}(^2G)]$
IV	2.44	2.21	2.24	2.54	2.22	2.24	$^4E_g(^4P)+^4A_{2g}(^4P)$[a]
V	-	-	2.53	-	-	2.66	[a]
VI	-	3.30	3.36	-	3.32	3.35	[a]

Feature	*(3)* experiment UV/Vis	*(3)* experiment RXES	*(3)* theory LFM convoluted states	*(4)* experiment UV/Vis	*(4)* experiment RXES	*(4)* theory LFM convoluted states	Assignment in D_{4h} without ζ_{3d}
I	-	0.31	0.26	-	0.33	0.29	$^4E_g(^4F)$
II	1.04	0.77	0.79	0.98	0.70	0.73	$^4E_g(^4F)+^4B_{2g}(^4F)$
III	2.14	-	1.49	-	-	1.33	$^4B_{1g}(^4F)$
IV	2.56	2.02	2.18	2.36	2.03	2.20	$^4E_g(^4P)+^4A_{2g}(^4P)$[a]
V	-	3.07	2.79	-	3.13	2.94	[a]
VI	-		3.18	-		3.60	[a]

[a] Group **IV**-**VI** consist of strongly mixed $^4E_g+^4A_{2g}(^4P)$, $^2A_{1g}(^2G)$, $^2E_g+^2A_{2g}(^2P)$, $^2E_g+^2A_{2g}+^2E_g+^2A_{2g}+^2E_g+^2B_{2g}+^2A_{1g}+^2B_{1g}(^2H)$ and $^2E_g+^2B_{2g}+^2A_{1g}+^2B_{1g}(^{2a}D)$ levels.

Comparison of *2p3d* RXES with UV/Vis

As mentioned in the main text, the spectral features in the UV/Vis spectra occur at higher energies than in the *2p3d* RXES spectra at excitation energy *d* for compound ***(1)*** to ***(4)***. The energy differences were quantified in Figure S4. There can be a number of causes for this shift in addition to experimental calibration errors as given in the main text. We have identified four possible causes, of which we addressed two with initial calculations.

The first effect is that the exact energy of a RXES peak may shift a little as a function of the excitation energy (for a fixed scattering and polarization geometry). It is not the energies of the manifolds that change, but the transition probability to them. To verify whether the difference originated from this effect, LFM calculated *2p3d* RXES spectra over the full *2p* XAS edge were calculated every 0.04 eV and summed. In the top of Figure S9 we show the experimental *2p3d* RXES spectrum at excitation energy *d* together with the UV/Vis spectrum of compound ***(1)*** again. On the bottom we show the LFM RXES spectrum at excitation energy *d* and the summed spectrum. While the summation broadens the features it does not explain the shift to higher energies in the UV/Vis spectrum.

The second effect relates to the polarization geometry of the experiment and calculations. All spectra that we showed in this article were LH polarized. This was done to increase the *2p3d* RXES signal as explained in the experimental section on page S6. We, and others before us,[4c, 4e] reported that a difference in polarization geometry may lead to changes in the energies of peak maxima. In Figure S9 we show a calculation for ***(1)*** at excitation energy *d* for a linear vertical (LV) polarized geometry (this geometry is experimentally accessible). We refer to our previous work for details on the experiment geometry and its calculation.[4e] It is visible in Figure S9 that a LV polarized spectrum has its energies at higher energy. The difference has been ascribed to the second order nature of the scattering process.[4c] We note that in Figure S9 all LFM calculations were normalized to their respective maxima, but that this gives a somewhat distorted picture of the relative intensities. For the LH and LV calculations, the former have an overall intensity (area under spectrum) that is ~4 higher at excitation energy *d*. How the spectra of different geometries should be combined to result at a decent comparison with the UV/Vis spectra remains a topic of further study. We simply want to indicate that this effect could be at the origin of the observed shifts.

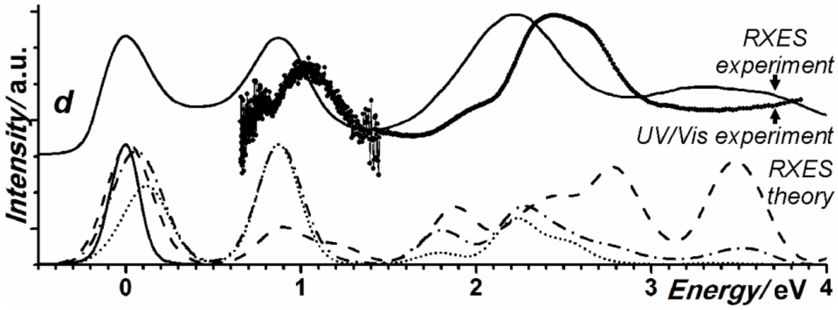

Figure S9. Top: experimental *2p3d* RXES spectrum at excitation energy *d* (solid line) and UV/Vis spectrum (dots+solid line) of ***(1)***. Bottom: the spectrum peaking at 0 eV (solid line) was taken off the sample to reveal X-ray scattering unrelated to the sample. LFM *2p3d* RXES spectra at excitation energy *d* (dotted line) and as summed over all energies of the *2p* XAS edge (dash-dotted line) for the LH polarization are shown. A LFM *2p3d* RXES spectrum at excitation energy *d* (dashed line) for the LV polarization is also shown.

Finally, we mention briefly two other possible causes for the energy shift that we cannot test with our LFM calculations. There are a number of ligand field manifolds in the RXES calculations that gain little intensity in the final spectra. These states are mostly double-electron or spin-forbidden states. They appear in the UV/Vis range and may possibly gain intensity in UV/Vis. However, since both double-electron and spin-forbidden d-d excitations are more allowed in *2p3d* RXES, this is not likely. In addition, for ***(4)*** the peak at 0.70 eV has no additional states in the calculation (as was shown in Table S3), yet it occurs at higher energies in UV/Vis. This makes the suggestion even more unlikely.

A fourth effect could be related to vibrational effects. In a simple model of UV/Vis absorption, vibrational side bands appear at higher energy. Because RXES is an emission spectroscopy, in first order the vibrational bands appear on the low-energy side. This would suggest that the UV/Vis peaks can be shifted to higher energy with respect to RXES peaks.

Alternative d-d sensitive techniques

Here we put our results in the context of four alternative techniques that measure d-d excitations. These techniques are electron energy loss spectroscopy (EELS),[14a] non-resonant inelastic X-ray scattering (NIXS),[14b] hard X-ray K pre-edge RXES[14c] and *3p3d* RXES.[14d] All references refer to NiO, except for the *3p3d* RXES reference. It is, as far as we know, the only system to which five different d-d sensitive techniques were applied. In principle, *2p3d* RXES has a resolution of 100 meV fwhm and the spectral variations over the *2p* XAS resonance provide additional information concerning the nature of the states. Hard X-ray K pre-edge RXES, EELS and NIXS show broader d-d features (250-300 meV fwhm) for various reasons. In addition, EELS and NIXS are not element-selective and thus not resonant, EELS is surface-sensitive and hard X-ray K pre-edge RXES is an electric quadruple excitation – electric quadruple decay experiment which has a low cross-section, implying long counting times. In addition, the published spectra have a large elastic peak which shadows the interesting region between 0.0 and 0.5 eV. This problem also occurs in *3p3d* RXES. The combination of good statistics at resonance, high resolution, detailed calculations and spectral variations over the XAS edge make *2p3d* RXES a very d-d sensitive tool.

References

The complete references of [3], [6], [8-10] and [13] are:

[3] a) G. Ghiringhelli, A. Piazzalunga, C. Dallera, G. Trezzi, L. Braicovich, T. Schmitt, V. N. Strocov, R. Betemps, L. Patthey, X. Wang, M. Grioni, *Rev. Sci. Instrum.* **2006**, *77*, 113108; b) V. N. Strocov, T. Schmitt, U. Flechsig, T. Schmidt, A. Imhof, Q. Chen, J. Raabe, R. Betemps, D. Zimoch, J. Krempasky, X. Wang, M. Grioni, A. Piazzalunga, L. Patthey, *J. Synchrotron Radiat.* **2010**, *17*, 631.

[6] S. M. Butorin, D. C. Mancini, J. H. Guo, N. Wassdahl, J. Nordgren, M. Nakazawa, S. Tanaka, T. Uozumi, A. Kotani, Y. Ma, K. E. Myano, B. A. Karlin, D. K. Shuh, *Phys. Rev. Lett.* **1996**, *77*, 574.

[8] *Other effects that in general may further split and shift the manifolds over a relatively small range of 0.1-0.2 eV, are the 3d spin-orbit coupling and magnetic superexchange interactions. In inorganic complexes the distance between metal ions is relatively large and the visible spectral effects of magnetic interactions (including collective ones) can be neglected.*

[9] *This holds for weak and strong ligand fields. It does not hold for the rare condition of very weak intra-atomic interactions (the peaks in region* ***B*** *would shift to region* ***A****).*

[10] *The weak ligand field implies that the lowest energy excitations could be one-electron excitations for* $3d^2$, $3d^3$, $3d^4$, $3d^6$, $3d^7$ *and* $3d^8$ *systems in all symmetries.*

[13] *We use the spectroscopic convention to put the initial state at the right side of a transition.*
